# Engineering Polyzwitterion and Polydopamine Decorated Doxorubicin-Loaded Mesoporous Silica Nanoparticles as a pH-Sensitive Drug Delivery

**DOI:** 10.3390/polym10030326

**Published:** 2018-03-15

**Authors:** Feng Ji, Hong Sun, Zhihui Qin, Ershuai Zhang, Jing Cui, Jinmei Wang, Shuofeng Li, Fanglian Yao

**Affiliations:** 1 School of Chemical Engineering and Technology, Tianjin University, Tianjin 300072, China; jifeng@tju.edu.cn (F.J.); zhihuiqin@tju.edu.cn (Z.Q.); eszhang@tju.edu.cn (E.Z.); wangjinmei067@163.com (J.W.); 2 Department of Basic Medical Sciences, North China University of Science and Technology, Tangshan 063000, China; 17710265395@163.com (J.C.); 15011337200@163.com (S.L.); 3 School of Pharmaceutical Science (Shenzhen), Sun Yat-sen University (SYSU), 135 Xingang Xi Road, Guangzhou 510275, China; 4 Key Laboratory of Systems Bioengineering of Ministry of Education, Tianjin University, Tianjin 300072, China

**Keywords:** mesoporous silica, surface modification, zwitterionic polymer, pH-sensitivity

## Abstract

Multifunctional drug carriers have great applications in biomedical field. In this study, we introduced both polydopamine (PDA) and zwitterionic polymer of poly(3-(3-methacrylamidopropyl-(dimethyl)-ammonio)propane-1-sulfonate) (PSPP) onto the surface of mesoporous silica nanoparticles (MSNs) to develop a novel nanoparticle (MSNs@PDA-PSPP), which was employed as a new kind of drug carrier for the delivery of doxorubicin (DOX). The PDA coating, as a gatekeeper, could endow the drug carrier with pH-sensitive drug release performance. The outermost PSPP layer would make the drug carrier possess protein resistance performance. The chemical structure and properties were characterized by Fourier transform infrared spectroscopy (FTIR), transmission electron microscopy (TEM), dynamic light scattering (DLS) and thermogravimetric analysis (TGA). MSNs@PDA-PSPP could keep good colloidal stability within 72 h in phosphate buffered saline (PBS) and protein solutions. Meanwhile, MSNs@PDA-PSPP exhibited a high drug loading for DOX. In vitro drug release experiments suggested MSNs-DOX@PDA-PSPP exhibited pH-dependent drug release behaviors. Besides, MSNs@PDA-PSPP had no cytotoxicity to human hepatocellular carcinoma cells (HepG2 cells) even at a concentration of 125 µg/mL. More importantly, cellular uptake and in vitro anticancer activity tests suggested that MSNs-DOX@PDA-PSPP could be taken up by HepG2 cells and DOX could be successfully released and delivered into the cell nuclei. Taken together, MSNs@PDA-PSPP have great potential in the biomedical field.

## 1. Introduction

Over the past decades, mesoporous silica nanoparticles (MSNs) have drawn great interest due to their unique properties such as high biocompatibility, easy synthesis and functionalization [1,2,3,4,5,6]. Additionally, MSNs possess a high loading capacity for small drugs, proteins and genes or co-delivery drugs, which is ascribed to their high surface area and large pore volume [7,8]. Normally, drugs enter into mesoporous channels mainly through adsorption. Considering the open pore structure of MSNs, the inherent premature leakage or uncontrolled release of the loaded drugs from MSNs is unwelcome, especially for the highly toxic drugs. Therefore, in order to reduce the premature leakage or uncontrolled release, capping the open pore by physical or chemical method to construct a gate-structure (named gatekeepers) is needed [9]. In order to realize the on-demand release of drugs, the gatekeepers are often designed with responsiveness associated with pathology, such as pH, temperature, redox potential and so forth. To date, various gatekeepers have been successfully prepared, such as macromolecule capping, polymer coating and nanomachine construction [10,11,12]. However, most of the gatekeepers still have some problems such as unpredictable toxicity or complicated process especially in the gate-sealing step. Thus, a gatekeeper with the merits of low cost, low risk, facile fabrication and stimuli-responsiveness is highly anticipated. One of the attracting biological features is the weak acidity of tumor sites. Typically, tumor tissues possess lower extracellular pH value (pH ca. 6.8) than normal tissues [13,14,15]. Based on this fact, pH-responsive gatekeepers have attracted special attention for the design and development of anti-cancer drug delivery carriers.

Luckily, an extremely rapid, one-step coating method for polydopamine (PDA) based on the oxidative self-polymerization of dopamine monomer in a weak alkaline condition (pH 8.0–8.5) has been widely used [16,17]. Being simple and versatile, PDA has been successfully coated on a wide range of inorganic, organic and biological materials for various applications [17,18,19]. Lots of studies have demonstrated PDA layer is sensitive to external pH [20,21,22,23,24]. With PDA layer on the surface of drug carrier, drug molecules can be easily blocked under neutral condition and released at lower pH value, which is due to the fact that the PDA layer can dissolve away when pH ≤ 5 [20,21,22,23,24]. Besides, PDA coating can react with nucleophilic compounds to further create new functional layers via Michael addition or Schiff base reactions. Many researchers have successfully used PDA coating as a secondary reaction platform to engineer various functional surfaces [23,24,25].

As everyone knows, when drug carriers come into blood, they often easily adsorb plasma proteins, which would lead to their clearance and further reduce the therapeutic outcome. In order to solve this problem, drug carriers must possess the ability to prevent protein adsorption. Zwitterionic polymers with equally cationic and anionic groups have been demonstrated to own excellent anti-protein adsorption property via electrostatically-induced strong hydration ability [26,27,28]. Typical zwitterionic polymers include poly(2-methacryloyloxyethyl phosphorylcholine) (PMPC), poly(sulfobetaine methacrylate) (PSBMA), poly(carboxybetaine methacrylate) (PCBMA) and so on [26,27,28]. Park et al. prepared dopamine coated poly(lactic-*co*-glycolic acid) (PLGA@PDA) nanoparticles and then used amine-terminated PCBMA to functionalize PLGA@PDA nanoparticles via “grafting to” method. Their results demonstrated PCBMA modified PLGA@PDA nanoparticles possessed good anti-protein adsorption performance [29].

Herein, the aim of this research was to engineer an anti-cancer drug carrier with high drug loading capability, pH-sensitive drug release performance and good anti-protein adsorption property. MSNs were selected as drug storage and PDA coating was served as gatekeeper. A zwitterionic polymer, thiol-terminated poly(3-(3-methacrylamidopropyl-(dimethyl)-ammonio)propane-1-sulfonate) (PSPP-SH) was decorated on the outside surface of MSNs@PDA by way of a Michael-type “thiol-ene” addition reaction, resulting in the formation of MSNs@PDA-PSPP. It could be predicted our engineered MSNs@PDA-PSPP would have high drug loading capacity due to the large surface area and pore volume of MSNs. The PDA coating, as a gatekeeper, could endow the drug carrier with pH-sensitive drug release performance. More importantly, the outermost PSPP layer would make the drug carrier possess good stability in the complex physiological environments because of its excellent anti-protein adsorption property. The size, size distribution, morphology, stability, drug loading content and in vitro drug release profiles of the prepared nanoparticles were investigated in detail. In vitro antitumor efficacy of MSNs-DOX@PDA-PSPP was assessed using HepG2 cell as the model cell.

## 2. Materials and Methods

### 2.1. Materials

3-[3-Methacrylamidopropyl-(dimethyl)-ammonio]propane-1-sulfonate (SPP) was synthesized according to a published report [30]. Dopamine hydrochloride, hexadecyl trimethyl ammonium bromide (CTAB), tetraethylorthosilicate (TEOS), 5,5′-dithiobis(2-nitrobenzoic acid) (DTNB), 4-cyano-4-(phenylcarbono-thioylthio) pentanoic acid (CTP) and 4,4′-azobis(4-cyanovaleric acid) (ACVA) were purchased from Aladdin (Shanghai, China). Doxorubicin hydrochloride (DOX) was purchased from Zhejiang Hisun Pharmaceutical Co. Ltd. (Taizhou, China). All other chemicals used were analytic grade.

### 2.2. Synthesis of PSPP and PSPP-SH

**Figure 1:**
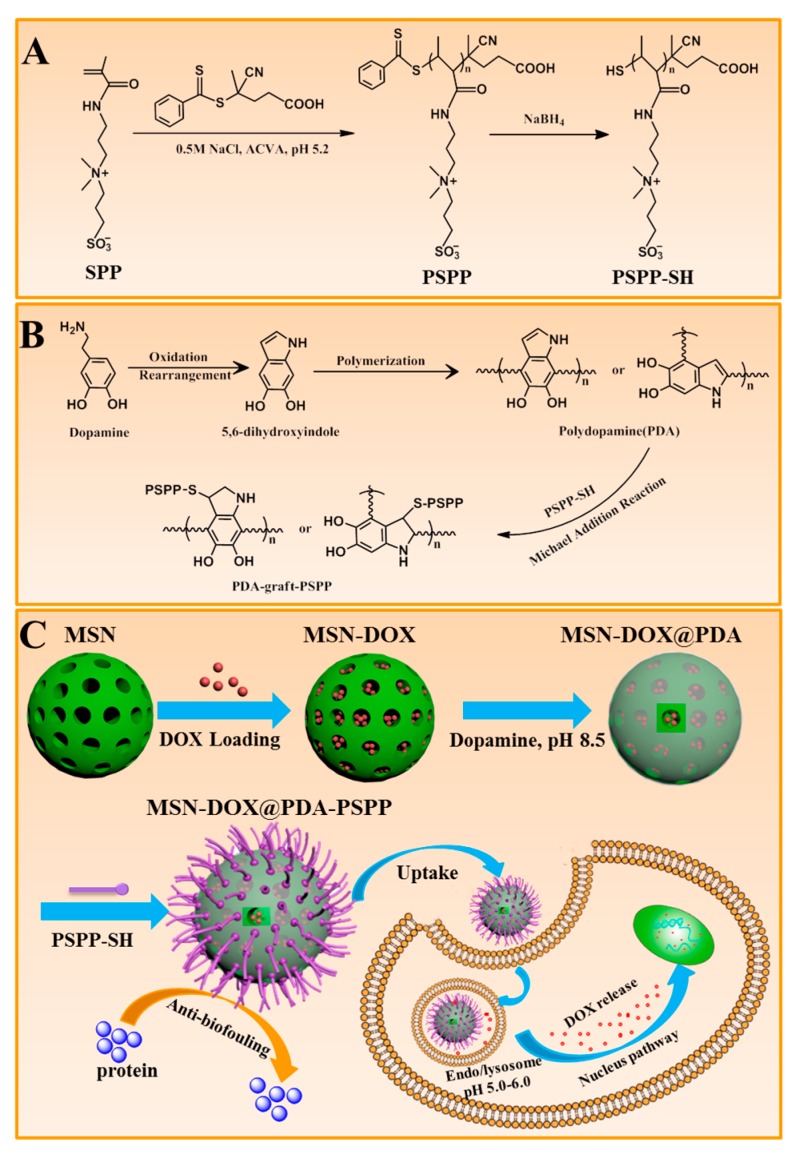
(**A**) Synthesis of poly(3-(3-methacrylamidopropyl-(dimethyl)-ammonio)propane-1-sulfonate) (PSPP-SH); (**B**) Oxidative polymerization of dopamine and conjugation of PSPP-SH with polydopamine (PDA) through Michael addition reaction; (**C**) Schematic illustration for the formation process of mesoporous silica nanoparticles-doxorubicin@PDA-PSPP (MSN-DOX@PDA-PSPP), the cellular uptake and drug release process of MSN-DOX@PDA-PSPP.

PSPP was synthesized by reversible addition-fragmentation-chain-transfer (RAFT) polymerization using CTP as RAFT chain transfer agent (Figure 1). In brief, CTP (16.76 mg, 0.06 mmol), SPP (0.35 g, 1.2 mmol) and ACVA (5.00 mg, 0.012 mmol) were weighed out into a Schlenk tube. Subsequently, 3 mL of 0.5 M NaCl solution was added and the pH value was adjusted to 5.2 using a NaOH solution (0.2 M). Next, the mixed solution was degassed via three freeze-vacuum-thaw cycles and placed in a pre-heated 70 °C oil bath. After 16 h, the polymerization was stopped by rapid cooling in an ice bath followed by dialysis in deionized water. Finally, PSPP (a pink solid) was obtained via lyophilization.

In order to obtain PSPP–SH, the dithioester end groups of PSPP were removed. In brief, 0.20 g of PSPP was dissolved in 2 mL of deionized water and the polymer solution was cooled to 0 °C, then 20 mg of sodium borohydride (NaBH_4_) was added and the reaction was carried out at 0 °C for 6 h. Subsequently, the reaction was quenched using 1 M HCl and the pH value of the solution was adjusted to ca. 3 to ensure the total completion of excess NaBH_4_. Then the solution was dialyzed against deionized water under N_2_ protection to avoid the oxidation of thiol groups. Finally, PSPP-SH (a white solid) was obtained by lyophilization and stored at −35 °C before use.

### 2.3. Preparation of MSNs

MSNs were prepared according to a previous report [31]. Briefly, CTAB (0.91 g) and NH_4_F (1.50 g) were dissolved in 250 mL of deionized water at 80 °C under mechanical stirring. Then, 4.5 mL of TEOS was added dropwise to the above solution and the resulting mixture was stirred for another 8 h. The white products (as-prepared MSNs) were obtained by centrifugation and washed with ethanol for three times. Finally, the product was dried under vacuum.

In order to remove the surfactant template (CTAB), the as-prepared MSNs were heated from 35 °C to 600 °C at a rising rate of 1 °C·min^−1^ and kept at 600 °C for 6 h.

### 2.4. Preparation of MSNs@PDA

MSNs@PDA were prepared by depositing PDA coating on MSNs via oxidative self-polymerization in a weak alkaline condition. Briefly, 80 mg of MSNs was added to the dopamine solution (40 mL, 1 mg/mL) in a Tris-HCl buffer (pH 8.5, 10 mM) and the polymerization was carried out for 8 h with constant shaking at room temperature. Afterwards, the resulting black products were centrifuged and washed with distilled water for several times. Finally, MSNs@PDA was obtained after lyophilization.

### 2.5. Conjugation of PSPP-SH to MSNs@PDA via Michael Addition Reaction

PSPP-SH was bound to the surface of MSNs@PDA via Michael addition reaction. In brief, 200 mg of PSPP-SH was added into 50 mL of MSNs@PDA solution (weak alkaline), then the mixture solution was stirred for 48 h at room temperature under N_2_ protection. The resulting MSNs@PDA-PSPP were collected by centrifugation and washed with distilled water.

### 2.6. Drug Loading and Release Studies

Doxorubicin hydrochloride (DOX) was used to investigate the drug loading and controlled release performance. DOX was loaded into MSNs through diffusion [31]. Briefly, 50 mg of MSNs were suspended in 5 mL of deionized water. Then doxorubicin hydrochloride (25 mg) was added and the mixture was stirred for 24 h in the dark. Next, the nanoparticles were collected by centrifugation and washed with neutral deionized water to remove the excess DOX. All the supernatants and washings were collected and then its absorbance was measured at 488 nm with a UV spectrophotometer. The weight of DOX loaded in MSNs was calculated according to the established calibration curve. DOX-loaded MSNs were designated as MSNs-DOX. The following preparation process of both MSNs-DOX@PDA and MSNs-DOX@PDA-PSPP was the same as the descriptions in Section 2.4 and Section 2.5, the corresponding products were designated as MSNs-DOX@PDA and MSNs-DOX@PDA-PSPP. The drug loading content (DLC) was calculated according to Equation (1).

(1) $$\mathrm{DLC}\left(\mathrm{wt}\;\%\right)=\frac{\mathrm{weight}\;\mathrm{of}\;\mathrm{DOX}\;\mathrm{in}\;\mathrm{the}\;\mathrm{nanoparticles}}{\mathrm{weight}\;\mathrm{of}\;\mathrm{nanoparticles}}\times 100\%\;$$

The release behaviors of DOX were investigated in PBS under a different pH. The detailed operation process was the same as our previous report [32]. Similarly, the cumulative release was calculated according to Equation (2). Where *W*_0_ (mg) was the weight of DOX in the drug carrier, *V*_1_ and *V*_2_ separately represented the volume of release medium and the withdrawn volume. The DOX concentration in the release medium for *n* and (*n*−1) times were separately denoted as *C_DOX(n)_* (mg/mL) and *C_DOX(n_*_−1)_ (mg/mL).

(2) $$\mathrm{Cumulative}\;\mathrm{release}\left(\%\right)=\frac{V_{1}C_{DOX\left(n\right)}+V_{2}\sum C_{DOX\left(n-1\right)}}{W_{0}}\times 100$$

### 2.7. Characterizations

The hydrodynamic size of the samples was measured using a NanoSizer Measurement (Malvern Instruments, Inc., Worcestershire, UK). The morphology of samples was observed via transmission electron microscope (TEM). For TEM sample preparation, 5 µL nanoparticle solution (0.5 mg/mL) was dropped onto a carbon membrane-coated copper grid. Before TEM characterization, the grid was allowed to dryness overnight at room temperature. The molecular weight and polydispersity index (*M_w_*/*M_n_*) of both PSBAA and PSBAA-SH were measured using gel permeation chromatography (GPC). An aqueous NaNO_3_ (0.1 M) solution was used as the eluent and poly(ethylene glycol) (PEG) with narrow molecular weight distribution was used as the standard for calibration. N_2_ adsorption-desorption isotherms were recorded at −196 °C using an ASAP 2020 accelerated surface area and porosity analyzer. Specific surface areas of the samples were determined from the adsorption data. Fourier transform infrared spectroscopy (FTIR) spectra of the samples were measured with a FTIR spectrometer. Thermogravimetric analysis (TGA) was carried out on a thermal analyzer under nitrogen (heating rate, 5 °C/min).

### 2.8. Cytotoxicity Evaluation of MSNs@PDA-PSPP

In vitro cytotoxicity of MSNs@PDA-PSPP was evaluated by CCK8 assay [33,34]. Briefly, HepG2 cells were seeded in a 96-well plate (4000 cells per well). Next, they were incubated for 24 h. Afterwards, the culture media was replaced by fresh media containing MSNs@PDA-PSPP at different concentrations (0, 7.8, 15.6, 31.2, 62.5 and 125 µg/mL). After 48 h, the cell viability was determined using CCK8 assay according to manufacturer’s instructions. The absorbance of each well was measured using an ELISA reader at 490 nm. The cell viability could be obtained based on Equation (3). Where the absorbance of the sample and control groups were separately denoted as *A_sample_* and *A_control_*. The cells without treatment with MSNs@PDA-PSPP was denoted as *A_control_*.

(3) $$\mathrm{Cell}\;\mathrm{viability}\left(\%\right)=\frac{A_{sample}}{A_{control}}\times 100\%\;\;$$

Furthermore, the acridine orange and propidium iodide (AO/PI) staining was conducted to further assess the cell cytotoxicity after 48 h of incubation with MSNs@PDA-PSPP. The cell morphology was observed by fluorescence microscopy.

### 2.9. Cellular Uptake Assay and Anticancer Activity Evaluation

The cellular uptake of MSNs-DOX@PDA-PSPP was researched by fluorescence microscopy [35,36]. In brief, HepG2 cells were seeded into 24-well cell cultured plates. After 24 h incubation, the cell culture media was replaced by fresh cell culture media containing free DOX or MSNs-DOX@PDA-PSPP. The concentration of DOX was 5 µg/mL or 10 µg/mL. After 2 h incubation, the cells were fixed using paraformaldehyde and the cell nuclei was stained with 4,6-diamidino-2-phenylindole (DAPI). After washing several times with PBS, the cell nuclei were analyzed using fluorescence microscopy with excitation wavelengths of 340 nm and the DOX was observed at excitation wavelengths of 485 nm.

The anticancer activity of MSNs-DOX@PDA-PSPP at different drug concentrations (0.05 µg/mL, 0.5 µg/mL, 5 µg/mL, 10 µg/mL and 40 µg/mL) was evaluated via CCK8 assay after 48 h incubation. The free DOX was used as the control.

### 2.10. Statistical Analysis

All data were expressed as mean ± SD. The differences between groups were assessed with a one-way ANOVA with Tukey’s posthoc test; a value * p < 0.05 was considered statistically significant.

## 3. Results and Discussion

The design and synthetic method of MSNs-DOX@PDA-PSPP were presented in Figure 1. Firstly, PSPP was synthesized by RAFT polymerization as it could precisely control the length of the desired polymer. Here, the reaction was carried out in 0.5 M NaCl solution because it was well known that electrolyte could enhance the water solubility of PSPP. The pH value of the reaction solution was performed at 5.2 to limit the hydrolysis of the dithiobenzoate agent. In order to obtain PSPP-SH, the dithioester end groups of PSPP were removed by NaBH_4_. Then MSNs were prepared and selected as drug storage because the size and pore volume of MSNs could be well controlled. Meanwhile, the large surface area and pore volume could endow MSNs with high drug loading capacity. Next, DOX was loaded into MSNs via diffusion in an aqueous media. Thereafter, PDA was attached to the surface of MSNs based on the spontaneous oxidative self-polymerization of dopamine monomer in a weak alkaline condition. The PDA coating not only took the role of gatekeeper, made MSNs-DOX@PDA exhibit sustained drug release behavior but also served as a secondary reaction platform. Finally, PSPP-SH was conjugated to the surface of MSNs-DOX@PDA via Michael addition reaction. The outermost layer of PSPP would make MSNs-DOX@PDA possess good stability in physiological environments due to its excellent anti-protein adsorption property.

### 3.1. Synthesis of PSPP and PSPP-SH

**Figure 2:**
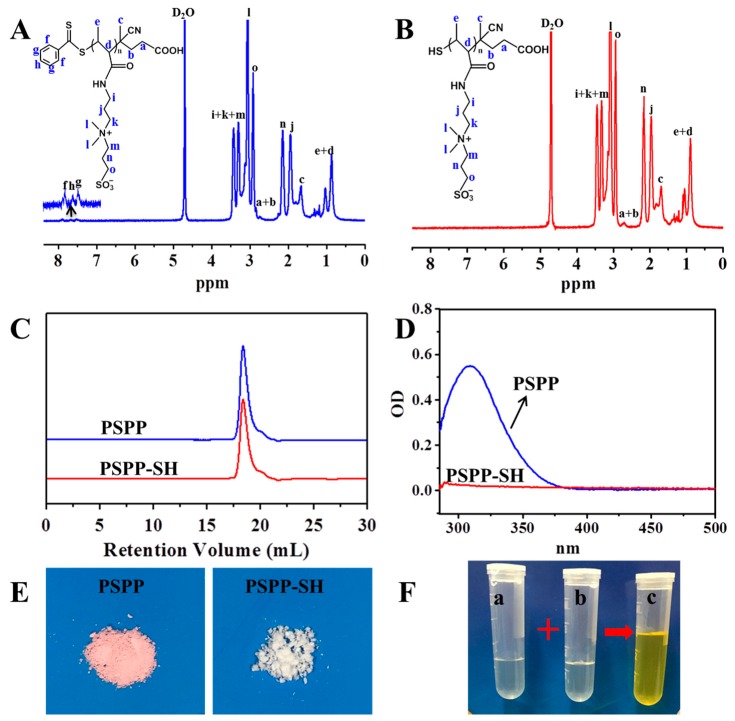
Characterization of PSPP and PSPP-SH. ^1^H NMR spectra of (**A**) PSPP and (**B**) PSPP-SH; (**C**) gel permeation chromatography (GPC) elution profiles (refractive index, R.I.). (**D**) UV spectra in deionized water at the polymer concentration of 0.25 mg/mL; (**E**) Digital photos of PSPP and PSPP-SH; (**F**) Digital photos of (c) Alman’s color reaction result of (a) 5,5′-dithiobis(2-nitrobenzoic acid) (DTNB) and (b) PSPP-SH in phosphate buffer (pH 8.0).

PSPP was synthesized by RAFT polymerization using CTP as RAFT chain transfer agent and ACVA as initiator for the first time (Figure 1A). The reaction was carried out in 0.5 M NaCl solution and the pH value of the reaction solution was performed at 5.2. The chemical structure of synthetic PSPP was confirmed by ^1^H NMR (Figure 2A). The polymerization degree of PSPP was 14, as calculated by comparing the integrals of the aromatic RAFT end-group signals (f, g and h) between 7.4 ppm and 8.0 ppm with the polymer side-chain signals at 3.07 ppm (assigned as l). Besides, the average molecular weight (M_n_) of PSPP was 3757 g/mol, as determined by GPC (Figure 2C). The GPC result was in agreement with that calculated from the ^1^H NMR (Figure 2A). The polydispersity index (PDI) of PSPP was 1.27, indicating RAFT polymerization of SPP monomer in 0.5 M NaCl solution at pH 5.2 was well-controlled. PSPP was purified by dialysis against deionized water and recovered by lyophilization to yield as a pink solid (Figure 2E).

In order to obtain PSPP–SH, the dithioester end groups of PSPP were reduced by NaBH_4_ (Figure 1A). As shown in Figure 2B, the disappearance of the aromatic RAFT end-group signals indicated that the dithioester successfully converted to thiol. In addition, the maximum UV absorbance at 309 nm of PSPP (Figure 2D) was attributed to the CTP dithioester end groups [37]. After reduced by NaBH_4_, the UV absorbance at 309 nm disappeared. These results indicated the successful preparation of PSPP–SH. Meanwhile, GPC analysis showed that the molecular weight and PDI of PSPP-SH remained almost unchanged with a unimodal distribution compared with PSPP. No bimodal distribution was observed, implying that disulfide coupling product (PSPP–S–S–PSPP) did not occur during the dialysis process. Furthermore, after reducing the end-group of PSPP, the color of the product changed from pink (the typical color of the thiobenzoate chain-end) to white (Figure 2E). Ellman’s reagent (DTNB) could react with free thiol groups in a phosphate buffer (pH 8.0) to generate 2-nitro-5-thiobenzoate. As 2-nitro-5-thiobenzoate was a chromophore and appeared yellow, thus DTNB was often used to qualitatively confirm the existence of thiol groups [38]. As expected, the color of PSPP-SH solution changed to yellow after mixing with DTNB (Figure 2F).

### 3.2. Preparation and Characterization of MSNs@PDA-PSPP

**Figure 3:**
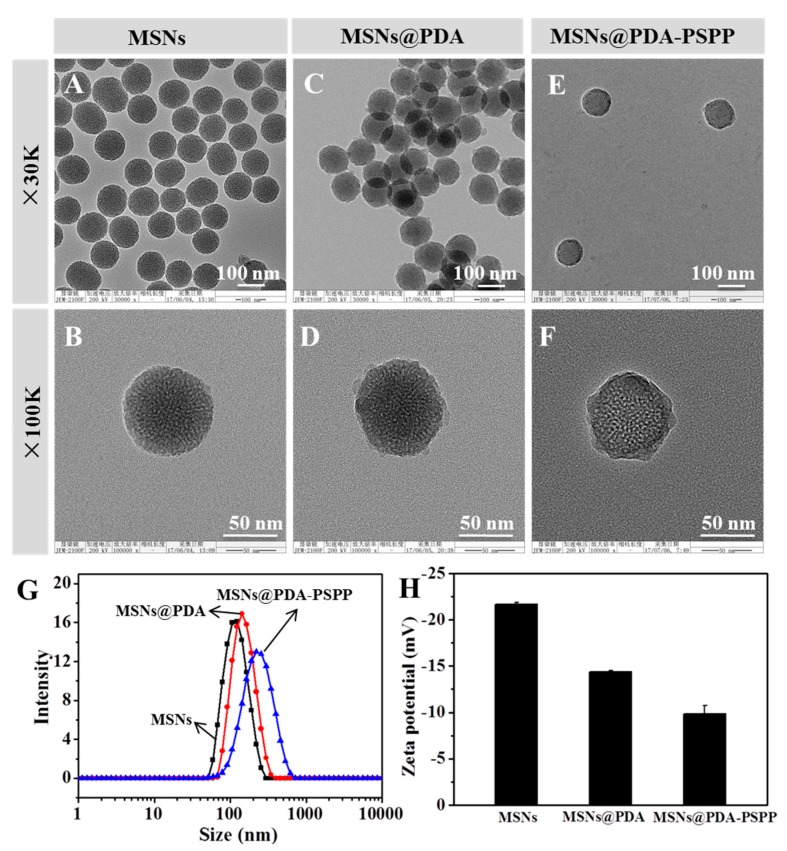
The morphology, size and zeta potential of MSNs, MSNs@PDA and MSNs@PDA-PSPP. The transmission electron microscopy (TEM) images of (**A**,**B**) MSNs, (**C**,**D**) MSNs@PDA and (**E**,**F**) MSNs@PDA-PSPP. The (**G**) average hydrodynamic size and (**H**) zeta potential of the different nanoparticles.

MSNs were prepared by the polycondensation of TEOS in the presence of CTAB and NH_4_F, followed by removal of the CTAB. First, the morphologies of MSNs and MSNs@PDA were observed by TEM. As shown in Figure 3A,B, MSNs displayed homogeneous and monodispersed spherical morphology. Meanwhile, MSNs' highly porous structure and the diameters of the well-organized hexagonal mesopores measured from the TEM images were about 2–3 nm. After coating PDA in Tris-HCl buffer (pH 8.5, 10 mM), a clear rough shell on the periphery of MSNs was observed (Figure 3C,D), which was an effective evidence for the successful formation of PDA coating layer on MSNs. Moreover, the sizes and size distributions of MSNs and MSNs@PDA were measured by DLS and the data were shown in Figure 3G. The size of MSNs was 111.4 ± 0.99 nm (PDI 0.134), while that of MSNs@PDA was 141.1 ± 0.96 nm (PDI 0.176). Comparing with MSNs, the size of MSNs@PDA increased about 30 nm, which was also regarded as an evidence of the successful coating of PDA. Meanwhile, the zeta potentials (Figure 3H) of MSNs and MSNs@PDA were also measured, the zeta potential of bare MSNs was −21.7 ± 0.2 mV due to the existence of negatively charged silanol groups. After surface modification with PDA, the zeta potential of MSNs@PDA increased to −14.4 ± 0.1 mV. The negative charge was attributed to the deprotonation of phenolic hydroxyl groups on PDA coating [22,24]. TEM images (Figure 3E,F) revealed that the MSNs@PDA-PSPP possessed excellent monodispersity. Compared with the size of MSNs@PDA, the size of MSNs@PDA-PSPP increased to 206.93 ± 2.02 nm (PDI 0.149) (Figure 3G), which was due to the strong hydration ability of PSPP. The zeta potential of MSNs@PDA-PSPP was −9.84 ± 0.89 mV (Figure 3H).

**Figure 4:**
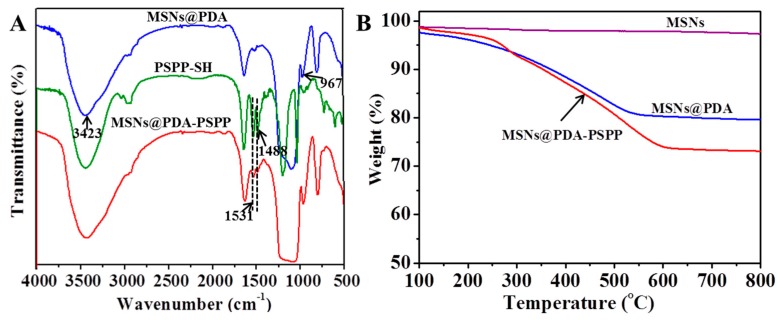
(**A**) Fourier transform infrared FTIR spectra of MSNs@PDA, PSPP-SH and MSNs@PDA-PSPP; (**B**) Thermogravimetric analysis (TGA) curves of MSNs, MSNs@PDA and MSNs@PDA-PSPP.

FTIR was carried out to confirm the chemical structure of the prepared nanoparticles (Figure 4A). For MSNs@PDA, the band at 967 cm^−1^ was the silanol group vibration. The broad absorbance at 3423 cm^−1^ was attributed to the stretching vibrations of N–H/O–H [22,24]. For PSPP–SH, the absorbance band at 1531 cm^−1^ was the symmetric stretching vibration of C–H in –CH_2_–N^+^ groups, while the band at 1488 cm^−1^ was assigned to the asymmetric stretching vibration of C–H in –N^+^(CH_3_)_2_ groups. Compared with MSNs@PDA, the new appeared adsorption band at 1488 cm^−1^ of MSNs@PDA-PSPP indicated PSPP-SH was successfully conjugated to the surface of MSNs@PDA.

Next, TGA was performed for quantitative analysis, as displayed in Figure 4B. The weight of bare MSNs from 100 to 800 °C was nearly constant, which indicated the surfactant CTAB was effectively removed by calcination during the preparation of MSNs. After surface functionalization of MSNs, the weight loss from 100 to 800 °C was 17.29 wt % for MSNs@PDA and 27.72 wt % for MSNs@PDA-PSPP, respectively, indicating that about 17.29 wt % of PDA and 10.43 wt % of PSPP was introduced onto the surface of MSNs. TGA results further demonstrated MSNs@PDA-PSPP were successfully prepared.

### 3.3. Stability of MSNs@PDA-PSPP

**Figure 5:**
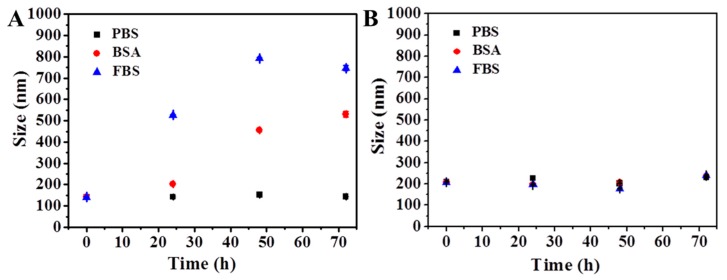
Size change of (**A**) MSNs@PDA and (**B**) MSNs@PDA-PSPP cultured in phosphate buffer saline (PBS), bovine serum albumin (BSA, 1 mg/mL) and 10% (*v*/*v*) fetal bovine serum (FBS) solution for 72 h.

For drug delivery carriers, good colloidal stability in biological condition was important because colloidal stability could influence the long circulation time and therapeutic efficiency. Normally, the size change of nanoparticles in PBS or protein solution was often used as a parameter to determine the stability of nanoparticles [31,39]. Thus, the size changes of MSNs@PDA and MSNs@PDA-PSPP in phosphate buffer saline (PBS), bovine serum albumin (BSA) and fetal bovine serum (FBS) solutions were measured. As shown in Figure 5A, MSNs@PDA were stable within 72 h in PBS solution due to the repulsion forces among the negatively charged nanoparticles [31,39]. However, in both BSA and FBS solution, we found the particle size increased within 24 h especially in FBS solution with the increase of incubation time, indicating lots of proteins adsorbed on the surface of MSNs. After 36 h incubation, precipitation was observed in the culture solution. The protein adsorption phenomenon of PDA coating was also observed and investigated by many researchers [25,40]. Lots of research results showed the adsorption behavior might arise from the adhesive nature of PDA coating and the attraction between the possible π–π stacking of the PDA layer and protein molecules [25,40]. As expected, the size of the obtained MSNs@PDA-PSPP nanoparticles nearly kept unchanged not only in PBS solution but also in protein media (Figure 5B), indicating few proteins could adsorb on the surface of MSNs@PDA-PSPP due to the excellent anti-protein adsorption ability of the PSPP layer. A tight hydration layer could form on the surface of MSNs@PDA-PSPP via ionic solvation interactions between PSPP moieties and water molecules, which could act as an energetic barrier to suppress the protein adsorption [27,28]. In summary, our prepared MSNs@PDA-PSPP had good colloidal stability in PBS and protein media.

### 3.4. Drug Loading and Release Behaviors

MSNs are widely used as drug delivery carriers because of their high loading capacity for various drugs, which is ascribed to their large surface area and pore volume. Here, we used MSNs as the drug storage for DOX. The specific surface area, pore volume and the most probable pore size of MSNs and MSNs-DOX were measured (Table 1). For bare MSNs, the Brunauer-Emmett-Teller (BET) surface area was 298.8 m^2^·g^−1^, the pore volume was 0.86 cm^3^·g^−1^ and the most probable pore size was 2.45 nm. After loading DOX, the BET surface area, the pore volume and the most probable pore size decreased to 145.1 m^2^·g^−1^, 0.51 cm^3^·g^−1^ and 1.93 nm, respectively. The BET result demonstrated DOX successfully occupied the mesoporous channels of MSNs.

**Figure 6:**
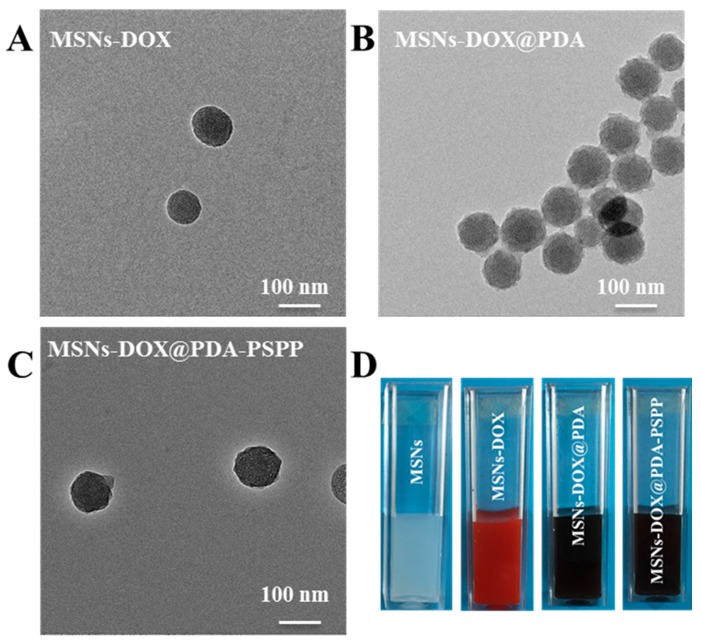
The TEM images of (**A**) MSNs-DOX; (**B**) MSNs-DOX@PDA and (**C**) MSNs-DOX@PDA-PSPP; (**D**) The digital photos of MSNs, MSNs-DOX, MSNs-DOX@PDA and MSNs-DOX@PDA-PSPP solution.

Furthermore, TEM analysis was performed to obtain the morphology of the DOX-loaded nanoparticles. As shown in Figure 6A–C, all the DOX-loaded nanoparticles (MSNs-DOX, MSNs-DOX@PDA and MSNs-DOX@PDA-PSPP) had a spherical morphology with excellent monodispersity, which was similar to that of the corresponding bare nanoparticles (MSNs, MSNs@PDA and MSNs@PDA-PSPP). The result also indicated DOX loading did not influence the dispersity of the nanoparticles. As shown in Figure 6D, MSNs were well dispersed in deionized water (uniform emulsions). After loading DOX, the color of MSNs-DOX solution was red. The final MSNs-DOX@PDA and MSNs-DOX@PDA-PSPP solution presented black.

The size, size distribution and DLC of DOX-loaded nanoparticles were shown in Table 2. We found the size and size distribution did not have great changes after DOX loading compared with the corresponding bare nanoparticles. The size of MSNs-DOX@PDA-PSPP was about 220 nm, which was appropriate for accumulating in tumor sites owing to the enhanced permeability and retention (EPR) effect. The DLC of MSNs-DOX, MSNs-DOX@PDA and MSNs-DOX@PDA-PSPP were 12.75 ± 0.42%, 9.86 ± 1.33% and 8.83 ± 1.19%, respectively.

**Figure 7:**
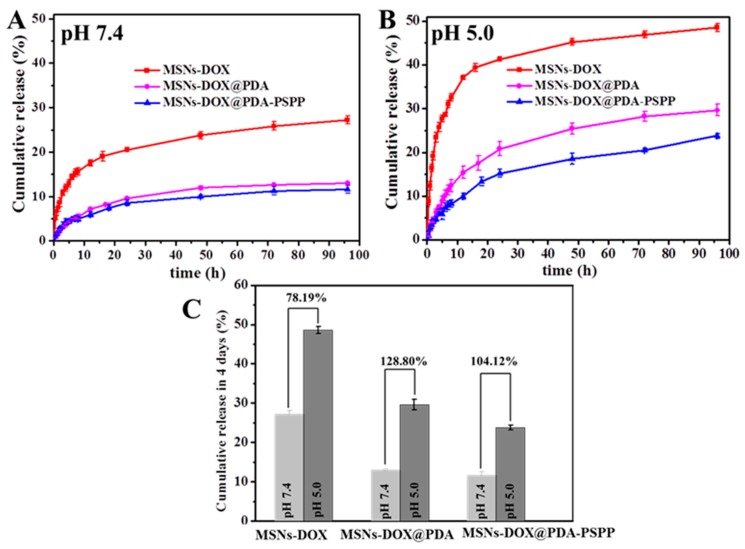
Cumulative release of DOX from MSNs-DOX, MSNs-DOX@PDA and MSNs-DOX@PDA-PSPP at (**A**) pH 7.4 and (**B**) pH 5.0; (**C**) The increase amplitude of drug release amount for MSNs-DOX, MSNs-DOX@PDA and MSNs-DOX@PDA-PSPP when the pH decreased from 7.4 to 5.0.

The DOX release behaviors of MSNs-DOX, MSNs-DOX@PDA and MSNs-DOX@PDA-PSPP were investigated at different pH (pH = 7.4, 5.0) for 96 h. As shown in Figure 7A,B, all the nanoparticles exhibited a typically biphasic release pattern: an initial quick release in the first 12 h and a subsequent sustained drug release for the rest time. In the physiological condition (pH = 7.4), about 27.24 ± 0.87% of DOX was released from MSNs-DOX over 4 days. Contrastingly, for MSNs-DOX@PDA and MSNs-DOX@PDA-PSPP, the amount of released DOX was 12.95 ± 0.18% and 11.65 ± 0.89%, respectively. The distinct release behaviors indicated the PDA coating and PSPP coating could effectively block the pores of MSNs and inhibit the drug release. The DOX release rate increased with the decrease of the pH value. When the pH value decreased from 7.4 to 5.0 with the purpose of simulating the environment of endosomes/lysosomes, both of MSNs-DOX@PDA and MSNs-DOX@PDA-PSPP exhibited a remarkably higher DOX release amount (29.63 ± 1.32% and 23.78 ± 0.55%, respectively). The conspicuous increase of DOX release of PDA-modified nanoparticles (MSNs-DOX@PDA, MSNs-DOX@PDA-PSPP) under acidic conditions suggested with the decrease of pH, the PDA coating on the surfaces might be partially removed, which unlocked the gate. Thus, more DOX was released from the channels of MSNs.

However, interestingly, it could be easily found with the decreasing of pH, the DOX release rate of MSNs-DOX also increased to 48.54 ± 0.88% from 27.24 ± 0.87%. The reason might be that acidic condition could improve the solubility of DOX, which was beneficial for the DOX release from drug carriers.

Besides, the increase amplitude of the drug release amount for the above drug carriers within 4 days was calculated when the pH decreased from 7.4 to 5.0 (Figure 7C). The increase amplitude for MSNs-DOX, MSNs-DOX@PDA and MSNs-DOX@PDA-PSPP was 78.19%, 128.80% and 104.12%, respectively. It could be easily observed the increase amplitude for MSNs-DOX@PDA and MSNs-DOX@PDA-PSPP was larger than that of MSNs-DOX, indicating PDA coating played its good role as a gatekeeper. On the other hand, the drug release amount of MSNs-DOX@PDA and MSNs-DOX@PDA-PSPP was smaller than that of MSNs-DOX within 96 h, implying PDA coating made the carriers possess better sustained drug release ability.

### 3.5. Cytotoxicity of MSNs@PDA-PSPP

**Figure 8:**
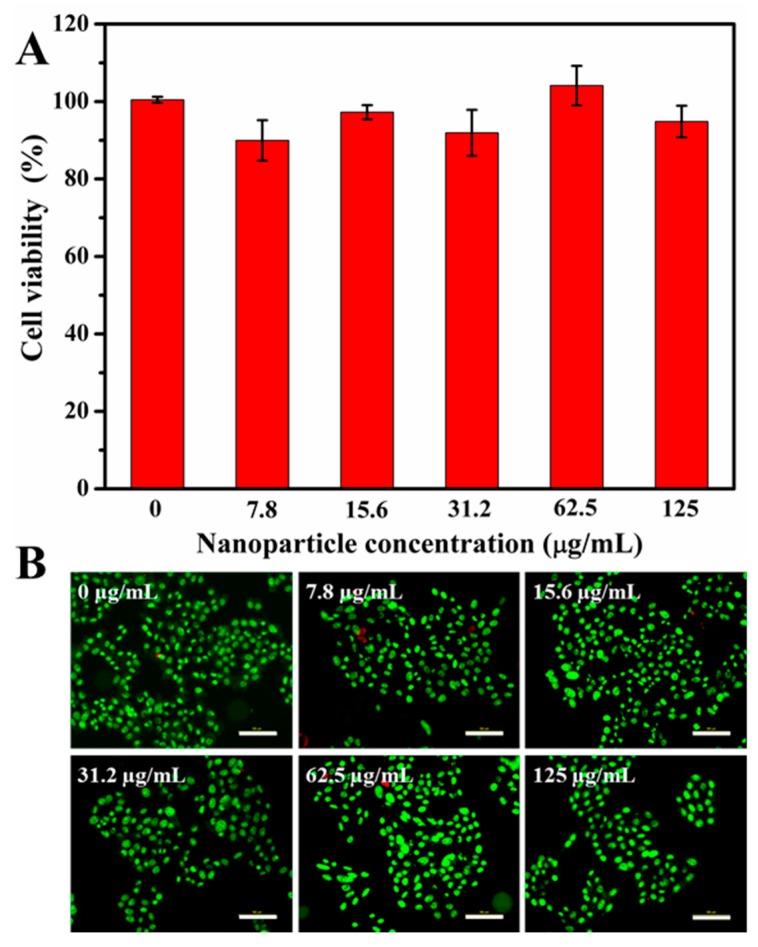
Cytotoxicity evaluation of MSNs@PDA-PSPP against HepG2 cells. (**A**) Cell viability of HepG2 cells after 48 h incubation with MSNs@PDA-PSPP at different concentrations, the data was expressed as mean ± SD, *n* = 3; (**B**) Fluorescence images of HepG2 cells stained with acridine orange and propidium iodide (AO/PI) after 48 h incubation at different concentrations (Green: live cells, red: dead cells). Scale bar = 100 μm.

The cytotoxicity of materials is an important parameter to determine whether they can be used as drug carriers [27]. Therefore, in vitro cytotoxicity of MSNs@PDA-PSPP against HepG2 cells was assessed via CCK8 assay and live/dead fluorescence staining. As shown in Figure 8A, the viability of HepG2 cells were above 90% (91.90–104.11%) after 48 h incubation at all tested concentrations (0–125 µg/mL). In addition, after stained with AO/PI, most of cells emerged green fluorescence (live cells) and few dead cells (red fluorescence) were observed (Figure 8B). These results indicated MSNs@PDA-PSPP had no obvious cytotoxicity to HepG2 cells even at the concentration of 125 µg/mL.

### 3.6. Cellular Uptake and Anticancer Effect

**Figure 9:**
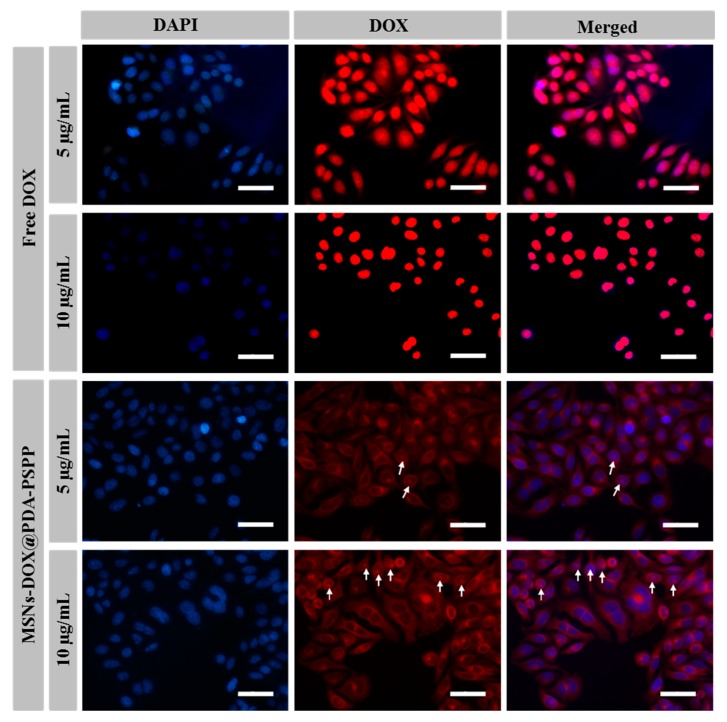
Fluorescence microscopy images of HepG2 cells after incubation with free DOX or MSNs-DOX@PDA-PSPP for 2 h at DOX concentrations of 5 µg/mL and 10 µg/mL. Scale bar = 50 µm. The white arrows indicated DOX successfully entered into the cell nuclei.

Normally, the internalization and drug release ability of drug carriers could influence the therapeutic outcomes. In this study, the cellular uptake of both free DOX and MSNs-DOX@PDA-PSPP were investigated via fluorescence microscopy. The cell nuclei showed blue fluorescence after stained with DAPI and DOX presented red fluorescence. As shown in Figure 9, we found after 2 h incubation, free DOX was mainly located in cell nuclei, which was due to the rapid diffusion mechanism of small molecules [27]. While, MSNs-DOX@PDA-PSPP was mainly located in the cytosol, suggesting MSNs-DOX@PDA-PSPP were internalized by endocytosis. Many other researchers also investigated the cell uptake of zwitterionic polymer-based drug carriers and demonstrated that the drug carriers were taken up by endocytosis [41,42,43]. When the drug concentration of MSNs-DOX@PDA-PSPP was 10 µg/mL, the red fluorescence (white arrows) could be also observed in cell nuclei, which indicated the loaded DOX was successfully released from MSNs-DOX@PDA-PSPP. 

Besides, the average gray values of different images were used as a criterion to compare the red fluorescent intensity and the result was presented in Figure S1. From Figure S1, we found HepG2 cells incubated with free DOX showed higher fluorescence intensity than that of MSNs-DOX@PDA-PSPP at the same condition, which was because free DOX and MSNs-DOX@PDA-PSPP had different cellular uptake mechanism [27,41]. Free DOX could quickly enter cells via passive diffusion and then immediately accumulate in the cell nuclei, while MSNs-DOX@PDA-PSPP were taken up by endocytosis. At the same incubation time, the red fluorescence intensity increased with the drug concentration for both free DOX and MSNs-DOX@PDA-PSPP.

**Figure 10:**
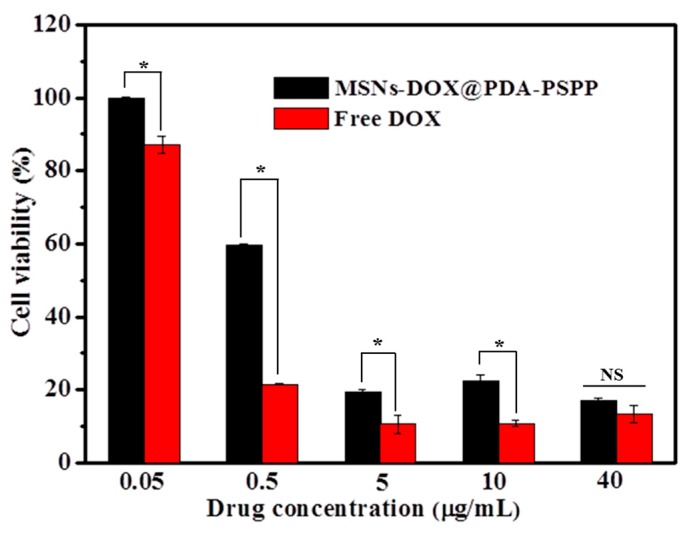
Cell viability of HepG2 cells after incubation with free DOX or MSNs-DOX@PDA-PSPP at different concentrations for 48 h (* p < 0.05).

In vitro anticancer activity of MSNs-DOX@PDA-PSPP against HepG2 cells was assessed by CCK8 assay. As shown in Figure 10, the cell viability of both free DOX group and MSNs-DOX@PDA-PSPP group exhibited a concentration-dependent effect. The cell viability decreased with the increase of drug concentration. Besides, MSNs-DOX@PDA-PSPP showed a lower anticancer effect than that of free DOX when the drug concentration was lower than 10 μg/mL, which might be caused by the slow release of DOX from the pore channels of MSNs-DOX@PDA-PSPP. When the drug concentration reached 40 μg/mL, free DOX and MSNs-DOX@PDA-PSPP showed similar anticancer activity. It must be noted although free DOX could effectively kill cancer cells in its free form, it had high toxicity to normal tissues/cells.

## 4. Conclusions

A novel pH-sensitive drug delivery carrier (MSNs@PDA-PSPP) was successfully prepared. The MSNs@PDA-PSPP could keep stable within 72 h in PBS, BSA and even FBS solutions. In vitro drug release experiment suggested MSNs-DOX@PDA-PSPP was sensitive to pH. The DOX release rate increased as pH decreased. CCK8 assay indicated MSNs@PDA-PSPP had no obvious cytotoxicity to HepG2 cells even at the concentration of 125 µg/mL. Moreover, MSNs-DOX@PDA-PSPP could be taken up by HepG2 cells and successfully release the loaded DOX to induce the cell death. In summary, our prepared MSNs@PDA-PSPP had great potential as drug carrier for cancer treatment.

## Figures and Tables

**Table 1:** BET analysis of MSNs and MSNs-DOX.

Nanoparticles	BET Surface Area (m^2^·g^−1^)	Pore Volume ^a^ (cm^3^·g^−1^)	Pore Size ^b^ (nm)
MSNs	298.8	0.86	2.45
MSNs-DOX	145.1	0.51	1.93

^a^ BJH cumulative pore volume for pores between 1.7 and 300 nm in width; ^b^ The most probable pore size.

**Table 2:** Characterization of DOX-loaded nanoparticles.

Nanoparticles	Size (nm)	PDI	DLC (%)
MSNs-DOX	123.3 ± 0.96	0.139	12.75 ± 0.42
MSNs-DOX@PDA	180.5 ± 1.20	0.106	9.86 ± 1.33
MSNs-DOX@PDA-PSPP	225.9 ± 3.12	0.182	8.83 ± 1.19

PDI = polydispersity index, DLC = drug loading content, *n* = 3.

## Supplementary Materials

The following are available online at http://www.mdpi.com/2073-4360/10/3/326/s1, Figure S1: Average gray value as a criterion to compare the red fluorescence intensity of different images in Figure 9.
