# Supplementary material for: Engineering Polyzwitterion and Polydopamine Decorated Doxorubicin-Loaded Mesoporous Silica Nanoparticles as a pH-Sensitive Drug Delivery
Source: Polymers (Basel). 2018 Mar 15;10(3):326. doi: 10.3390/polym10030326 (PMC6415439; doi:10.3390/polym10030326)
Supplement: Supplementary file 1 [file polymers-10-00326-s001.pdf]

Supplementary Materials:

Engineering polyzwitterion and polydopamine decorated doxorubicin-loaded mesoporous silica nanoparticles as a pH-sensitive drug delivery

Feng Ji [1], Hong Sun [2,*], Zhihui Qin [1], Ershuai Zhang [1], Jing Cui [2], Jinmei Wang [1,3], Shuofeng Li [2] and Fanglian Yao [1,4,*]

1 School of Chemical Engineering and Technology, Tianjin University, Tianjin 300072, China; jifeng@tju.edu.cn (F.J.); zhihuiqin@tju.edu.cn (Z.Q.); eszhang@tju.edu.cn (E.Z.); wangjinmei067@163.com (J.W.);

2 Department of Basic Medical Sciences, North China University of Science and Technology, Tangshan 063000, China; 17710265395@163.com (J.C.); 15011337200@163.com (S.L.);

3 School of Pharmaceutical Science (Shenzhen), Sun Yat-sen University (SYSU), 135 Xingang Xi Road, Guangzhou, 510275, Guangdong, P. R. China;

4 Key Laboratory of Systems Bioengineering of Ministry of Education, Tianjin University, Tianjin, 300072, China;

* Correspondence: yaofanglian@tju.edu.cn (F.Y.); 3725930@163.com (H.S.);

Results and Discussion

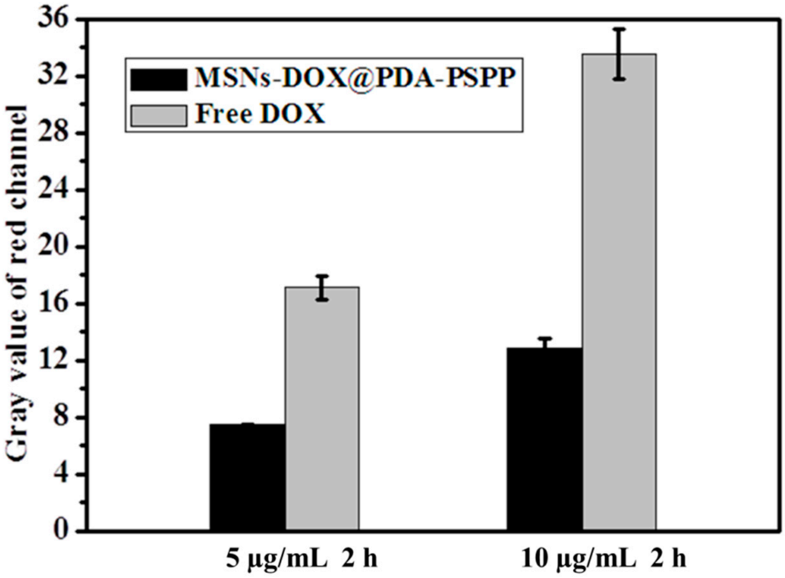

Figure S1. Average gray value as a criterion to compare the red fluorescence intensity of different images in Figure 9.
